# Segmental reversal of the distal small intestine in a short bowel syndrome model in piglets showed detrimental effect on weight gain

**DOI:** 10.1186/s12876-022-02418-3

**Published:** 2022-07-20

**Authors:** Lasse Hartmann Schmidt, Jesper Stensig Aa, Bolette Hartmann, Gunvor Iben Madsen, Niels Qvist, Mark Bremholm Ellebæk

**Affiliations:** 1 Research Unit for Surgery, Odense University Hospital, University of Southern Denmark, J. B. Winsløws Vej 4, 5000 Odense C, Denmark; 2 Department of Medical Sciences, NNF Center for Basic Metabolic Research, Faculty of Health Science, University of Copenhagen, Blegdamsvej 3B, 2200 Copenhagen N, Denmark; 3 Research Unit for Pathology, Odense University Hospital, University of Southern Denmark, J. B. Winsløws Vej 4, 5000 Odense C, Denmark

**Keywords:** Short bowel syndrome, Short gut syndrome, Segmental reversal, GLP-2, GIP

## Abstract

**Background:**

To investigate the effects of a reversed segment of the distal small intestine to improve weight gain in an experimental short bowel syndrome (SBS) model in piglets.

**Methods:**

Twenty-four piglets underwent resection of 70% of the distal small intestine. In half of the animals a conventional anastomosis was performed, and in the other half, the distal 25 cm of the remnant jejunum was reversed before the intestinal continuity was recreated. Weight was measured daily until day 28, where the animals were euthanized. Glucagon-Like Peptide-2 (GLP-2) and Glucose-dependent Insulinotropic Peptide (GIP) was measured pre- and postoperatively at day 28.

**Results:**

The group with reversal of small intestine had a significant lower weight gain at 5.26 ± 3.39 kg (mean ± SD) compared to the control group with 11.14 ± 3.83 kg (p < 0.05). In the control group greater villus height and crypt depth was found distally, and greater muscular thickness was found proximally in the intervention group. GLP-2 and GIP levels increased significantly in the control group.

**Conclusions:**

Treatment of short bowel syndrome with a reversed jejunal segment of 25 cm had a detrimental effect on the weight gain.

## Background

Intestinal failure is defined by the inability to maintain normal metabolism of fluids, electrolytes, micronutrients, and proteins enterally [1]. Short bowel syndrome (SBS) is one reason among several others for intestinal failure. SBS is often a result of extensive resection of the small intestine from complications to surgery, inflammatory bowel disease and congenital intestinal malformations [2, 3]. The syndrome is characterized by diarrhoea, malnutrition and weight loss, and the need for, parenteral nutrition (PN) [4]. Patients dependent of home parenteral nutrition (HPN) due to SBS have a 5 year survival at 72%, but death directly related to HPN is rare [5, 6]. The survival is usually determined by the underlying disease [5]. SBS and dependency of HPN is associated with impaired quality of life and an economic burden for the healthcare system [5, 7, 8].

Two different surgical options to improve intestinal absorption are a lengthening procedure or reversal of an intestinal segment. The Bianchi procedure and Serial Transverse EnteroPlasty (STEP) are both lengthening methods, and most often used in children with congenital malformations. The Bianchi method is technically demanding and the STEP method are only applicable in patients who have developed intestinal dilatation. The method with segmental reversal have primarily been utilized in patients in connection with a stoma reversal operation. The segmental reversal may increase gastrointestinal transit time by retrograde peristalsis, and thereby improve absorption of fluid and nutrients [9].

The study by Beyer-Berjot et al. [7] showed that 17 out of 38 patients could be weaned of PN after the segmental reversal procedure on the distal part of the small intestine, and in the other 21 patients the number of days on intravenous infusion was reduced from 7 to 4 days a week. The length of the reversed segment was between 6 and 15 cm [7]. No systematic studies have been performed regarding the optimal length of the reversed segment to obtain maximal nutritional effect.

Glucagon-Like Peptide-2 (GLP-2) is secreted from L-cells located in the distal ileum and colon in response to enteral feeding [10]. GLP-2 may contribute in the improved absorption, as it induces crypt cell proliferation, prevents enterocyte apoptosis and inhibits intestinal motility [11–13]. Exogenous GLP-2 has shown to improve absorption of nutrients and to increase weight gain in both animal SBS models [14, 15] and in patients with SBS [16]. Glucose-dependent Insulinotropic Peptide (GIP) is secreted from the enteroendocrine K-cells in the duodenum when exposed to food and stimulate insulin release[17]. To our best knowledge the endogenous hormonal levels of GLP-2 and GIP after a segmental reversal have yet to be investigated.

The primary aim was to determine the weight gain at day 28 after reversal of a 25 cm small intestine segment in a SBS model in piglets. Secondary aims was to investigate the effects on histological trophic changes in the intestine and changes in GLP-2- and GIP-levels in peripheral blood.

## Methods

### Study design

Non-blinded randomized experimental animal study. The animals were randomized to a control group (n = 12) or an intervention group (n = 12) (http://www.random.org). SBS were induced by resection of 70% of the distal small intestine in both groups. In the intervention group a 25 cm long segment of the distal small intestine was reversed 180 degrees. At the end of the study, 28 days after surgery, all piglets were pre-anesthetized as described, a blood sample was collected and hereafter the animals were sacrificed.

### Animals

24 weaned female piglets (Species: *Sus scrofa domesticus*, Landrace × Yorkshire) of approximately six weeks of age and median weight of 16.5 kg (range 10.9–18.8) were included. The piglets were obtained from a conventional local farm at Funen, Denmark, housed under standardized conditions, with automatized temperature and light, access to a heating lamp and, free access to water and forage. Preoperative fasting started 12 h before surgery. One piglet in the intervention group was euthanized at postoperative day 8 due to volvulus and was excluded from the study.

### Sample size estimation

A difference in weight gain between the two groups of piglets of at least 20% was considered clinically significant. With a standard deviation of 2.4, a significance level of 0.05, and a power of 80% this study will require the inclusion of 11 piglets in each group. With an expected mortality of 10%, a total of 24 piglets were needed.

### Anaesthesia

Pre-anaesthesia comprised a combination of midazolam 0.2 mg/kg (Hameln Pharma, Hameln, Germany), medetomidinhydrochlorid 0.04 mg/kg (Cepetor Vet, CP-Pharma Handelsges. GmbH, Burgdorf, Germany) and atropin 0.05 mg/kg (Takeda Pharma A/S, Taastrup, Denmark) administered intramuscularly. Anaesthesia was induced with propofol 5 ml/10 kg (B. Braun Melsungen AG, Melsungen, Germany) administered through an ear vein. Preceding intubation (cuffed tube size 5.0, Rüschelit®, Super Safety Clear, Teleflex Medical Inc., Athlone, Ireland) local anaesthesia was sprayed onto the vocal cords (Xylocaine®, 10 mg/dose, Paranova Danmark A/S, Herlev, Denmark). Anaesthesia was maintained with isoflurane, 2.2% in oxygen/air (2:1) using a ventilator (Dameca MCM 801, Rødovre, Denmark) at a respiratory frequency of 16 per minute and a tidal volume of 10 ml/kg. Continuous blood pressure, electrocardiogram, heart rate and oxygen saturation were monitored. Per-operative analgesia comprised of intravenous administration of fentanyl 50 µg/kg/h (B. Braun Melsungen AG, Melsungen, Germany).

### Surgical procedures

The small intestine was exposed through a 10 cm lower midline laparotomy. The ileocecal ligament was identified and the total length of the small intestine to the ligament of Treitz was measured at the anti-mesenteric border, with a sterile flexible liner. Twenty cm orally to the ileocecal ligament in proximal direction, 70% of the total length of the small intestine was resected. In the intervention-group the distal 25 cm of the remaining jejunum was reverted 180 degrees. Prior to re-establishment of intestinal continuity, the anastomoses (two in the intervention-group and one in the control group) were performed end-to-end with a running single-layer seromuscular suture Monocryl 4- 0 (Ethicon®, Johnson & Johnson International, Diegem, Belgium). The abdominal fascia was closed with a PDS-0 (Ethicon®, Johnson & Johnson International, Diegem, Belgium) and the skin intracutaneously with a running Monocryl 2-0 (Ethicon®, Johnson & Johnson International, Diegem, Belgium). Finally, the wound was sealed with OPSITE® (Smith & Nephew, London, United Kingdom). Total length of the intestine, intestine removed and remaining small intestine is shown in Table 1.

**Table 1:** Characteristics of the intervention and control group

Variable	Intervention group, n = 11	Control group, n = 12	*p*
Mean (range) kg			
Starting weight	16.23 (10.9–18.5)	15.82 (11.2–18.8)	0.6669
Mean (range) cm			
Small intestine length	759.1 (550–1185)	749.6 (595–915)	0.5626
Small intestine removed	538.23 (385–905)	524.71 (416.5–640.5)	0.6131
Small intestine remaining	220.86 (165–291)	224.88 (178.5–274.5)	0.2819

Characteristics of weight and intestinal length between the intervention group (Short bowel syndrome with reversal) and control group (Short bowel syndrome)

### Post-operative management

The piglets had free access to water and forage (Powder forage: Nutrimin Recept 62252825-I, Ans By, Denmark. Pellets: Porkido Plus Start BFU, DLG, Denmark. Treats: Special Diets Services, Essex, England). Postoperative pain management consisted of a fentanyl patch 2 µg/kg/h (Matrifen®, Takeda Pharma A/S, Taastrup, Denmark) for the first 6 days, and applied immediately after surgery and changed day 3 post-operatively. Butorphanol 0.2 mg/kg (Dolorex®, Intervet International B.V., Boxmeer, Netherland) and metadonhydrochlorid 0.2 mg/kg (Comfortan® Vet., Dechra, Shrewsbury, United Kingdom) was administered intramuscularly every 4th hour for the first 24 h post-operatively.

Weight was measured daily.

### Antibiotic prophylaxis

Metronidazole 20 mg/kg (B. Braun Melsungen AG, Melsungen, Germany) was administered intravenously 30 min before surgery, and metronidazole 20 mg/kg (Flagyl®, Sanofi-Aventis A/S, Hørsholm, Denmark) given once a day orally in the first five days postoperative. Amoxicillin 15 mg/kg (Curamox® Prolongatum vet, Boehringer Ingelheim A/S, Copenhagen, Denmark) was administered intramuscularly after 48 and 96 h.

## Blood samples

Samples for the analysis of GLP-2 and GIP were collected on day 0 and 28, in EDTA tubes containing 30µL dipeptidyl peptidase-4-inhibitor (BD Vacutainer® K2E (EDTA) 7.2 mg with Valine Pyrolidide) to avoid the degradation of GLP-2. The tubes were centrifuged at 2000 RPM at 4 °C for ten minutes. Plasma was pipetted into microtubes and stored at − 80 °C. All samples were extracted in a final concentration of 75% ethanol before GLP-2 measurements and 70% ethanol before GIP measurements. Intact GLP-2 was measured using an in-house developed radio-immuno assay as previously described [18]. The antiserum (Code No. 92,160) is directed against the N-terminus of GLP-2 and therefore measures only fully processed GLP-2 of intestinal origin. For standards, we used recombinant human GLP-2 and the tracer was ^125^I-labeled rat GLP-2 with an Asp33 -> Tyr33 substitution.

GIP was measured using a C-terminally directed antibody (Code No. 80867), which reacts fully with intact GIP and N-terminally truncated forms as previously described [19]. The standard was human GIP (Bachem, Cat No. H-5645) and the tracer was ^125^I-labeled human GIP (Perkin Elmer, Cat no. Nex402). Sensitivity for both assays was below 5 pmol/l, and intra assay coefficient of variation below 10%.

### Euthanasia

The piglets were pre-anesthetized as described previously, and an ear vein catheter was placed. A dose of 140 mg/kg of pentobarbitalnatrium (Euthasol vet., Le Vet B.V., Oudewater, The Netherlands) was administered intravenously for euthanizing.

### Macroscopical and histopathological analysis

At autopsy the abdomen was inspected for abnormalities including adherences, leakage, and abscesses. Any dilation of the intestine was assessed visually. For histological analysis a short segment (2 cm) of the small intestine was excised 5 cm proximally and distally to the anastomosis in the control group, and in the intervention group proximally to the proximal anastomosis, distally to the distal anastomosis, and in the middle of the reversed segment. The samples were fixated in a 4% paraformaldehyde solution. Within 24–72 h, the samples were dehydrated and infiltrated with paraffin and cut in 3-µm slices and stained with hematoxylin and eosin.

The slices were scanned with a Nanozoomer 2.0 HT® (Hamamatsu Photonics, Hamamatsu City, Shizuoka Pref., Japan). Villus height and crypt depth was measured at ten different locations, thickness of circular and longitudinal muscle-layer at five different locations, and the median was used for statistical analysis.

For the immunohistochemical staining, sections of two µm sclices were cut and mounted on FLEX IHC slides (Dako, Glostrup, Denmark). Slices were dried at room temperature and baked at 60 °C for 20 min before immunostaining. Staining was automated at the BenchMark Ultra immunostainer (Ventana Medical Systems, Tucson, Arizona, USA) using the OptiView-DAB detection (Ventana Medical Systems, Tucson, Arizona, USA). Incubation with villin, clone CWWB1 (Ventana Medical Systems, Tucson, Arizona, USA), was done for 32 min at 36 °C. Epitope retrieval was performed in Cell Conditioning Solution 1 (CC1) for 48 min at 100 °C. Incubation with Ki-67, clone 30−9 (Confirme Rabbit Monoclonal, Ventana Medical Systems, Tuscon, Arizona, USA), was done for 12 min at 36 °C. Epitope retrieval was performed in Cell Conditioning Solution 1 (CC1) for 48 min at 100 °C. The nuclear counterstaining in both villin and Ki-67 specimens were performed using Hematoxylin II (Ventana Medical Systems, Tucson, Arizona, USA). Finally, slides were washed, dehydrated, and coverslipped using an Agilent/Dako Coverslipper.

The staining with Ki-67 and villin was graded as “1” (lightly stained), “2” (moderately stained) and “3” (densely stained). The level of proliferation was shown using Ki-67. The zone of proliferation dictated the grading. Proliferation at the bottom of the mucosal layer was graded “1”, proliferation in an expanded zone as grade “2”, and highly increased proliferation in the whole mucosal layer as grade “3”. A blinded experienced pathologist performed the measurement and grading.

### Statistical analysis

The two-sample student’s *t*-test was used to compare the difference in weight gain between the two groups, and Mann–Whitney-*U* test or Wilcoxon signed-rank test for the analysis of GLP-2 and GIP levels, histopathological measurements and immunohistochemical grading. Regression analysis was performed with the difference in weight gain and GLP-2 and GIP-levels as an outcome and preoperative weight and the remaining length of the small intestine as predictors. *P *values < 0.05 were considered statistically significant. Statistical analyses were performed using Stata IC version 15.1; Texas, USA.

## Results

### Weight gain

**Fig. 1:**
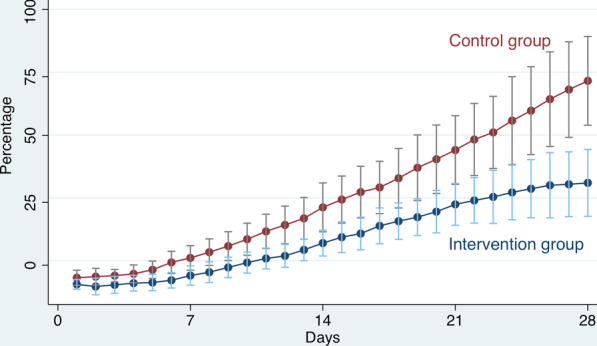
Percentage weight gain. Percentage weight gain and 95% CI for the intervention group with short bowel syndrome and segmental reversal, and for the control group with short bowel syndrome

The intervention group with segmental reversal gained significantly less weight measured at postoperative day 28 compared with the control group. The mean values and SD were 5.26 ± 3.39 kg and 11.14 ± 3.83 kg respectively (p = 0.0004), corresponding to a weight gain of 31.0 ± 19.8% and 71.6 ± 27.8% (p = 0.0003) as shown in Fig. 1.

The regression analysis regarding weight gain showed no significant difference, with starting weight and length of the small intestine as predictors.

### Histology

Villus height was significantly higher in the control group both in the proximal (451.5 μm, 397.0; 602.0) (Median, IQR 25; 75) and distal segment (557 μm, 453.5; 700.5) compared to the intervention group (365 μm, 315.0; 394.0 and 388 μm, 340.0; 460.0 respectively). Crypt depth was significantly greater in the distal segment in control animals (255 μm, 219.0; 279.5) compared to the intervention group (193 μm, 159.0; 260.0). The intervention group with distal segmental reversal had significantly greater circular (572 μm, 521.0; 638.0)—and longitudinal (300 μm, 225.0 ; 427.0) muscle thickness in the proximal segment compared to controls (290 μm, 218.0; 315.5 and 167.5 μm, 144.0; 217.0 respectively), but not in the distal segment. Villin and Ki-67 staining showed no significance between the two groups (Table 2).

**Table 2:** Histological results

Median (IQR 25;75) µm	Intervention group, n = 11	Control group, n = 12	*p*
**Proximal segment**			
Villus height	365.0 (315.0; 394.0)	451.5 (397.0; 602.0)	0.0038*
Crypth depth	173.0 (162.0; 220.0)	207.0 (183.5; 223.5)	0.1163
Circular muscle thickness	572.0 (521.0; 638.0)	290.0 (218.0; 315.5)	0.0031*
Longitudinal muscle thickness	300.0 (225.0; 427.0)	167.5 (144.0; 217.0)	0.0074*
Villin	3.0	3.0	
Ki-67	2.0	1.0	0.0596
**Reversed segment**			
Villus height	355.0 (313.0; 393.0)		
Crypth depth	176.0 (139.0; 267.0)		
Circular muscle thickness	380.0 (324.0; 472.0)		
Longitudinal muscle thickness	228.0 (172.0; 261.0)		
Villin	3.0		
Ki-67	2.0		
**Distal segment**			
Villus height	388.0 (340.0; 460.0)	557.0 (453.5; 700.5)	0.0097*
Crypth depth	193.0 (159.0; 260.0)	255.0 (219.0; 279.5)	0.0312*
Circular muscle thickness	270.0 (204.0; 324.0)	308.5 (175.0; 388.5)	0.8535
Longitudinal muscle thickness	152.0 (134.0; 255.0	184.5 (124.0; 258.5)	0.9754
Villin	3.0	3.0	
Ki-67	2.0	2.0	0.8857

Histological results for the intervention group (Short bowel syndrome with reversal) and control group (Short bowel syndrome)

*Statistical significant value (*p* < 0.05)

### GLP-2 and GIP-levels

**Fig. 2:**
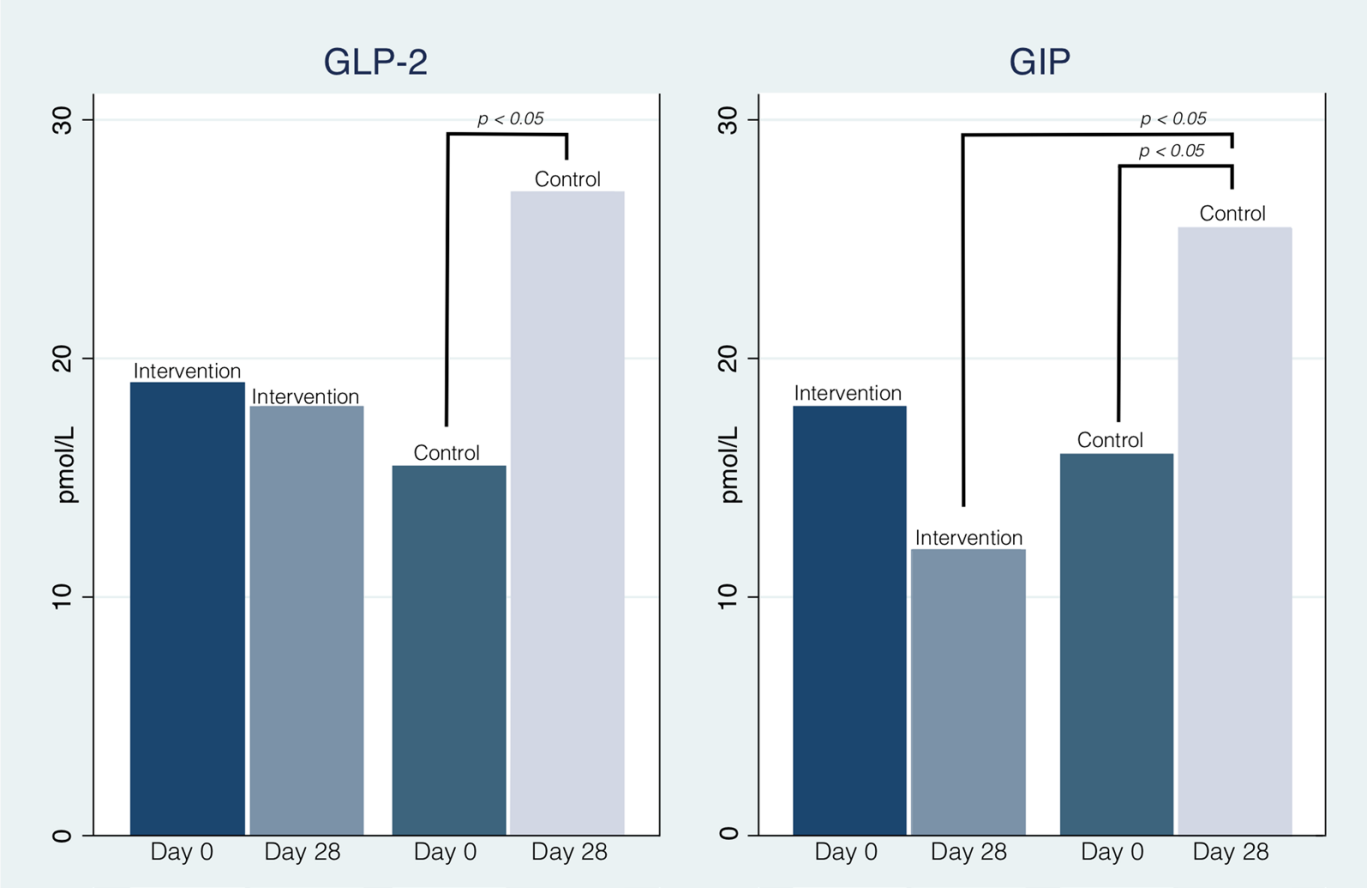
GLP-2- and GIP-levels. Median levels of Glucagon-Like Peptide-2 (GLP-2) and Glucose-dependent Insulinotropic Peptide (GIP) on day 0 and 28 for the for the intervention group with short bowel syndrome and segmental reversal, and for the control group with short bowel syndrome

No significant difference in GLP-2 and GIP-levels was found in the intervention group measured at day 0 and day 28, whereas levels for both hormones increased significantly in the control group. GIP-levels were significantly lower in the intervention group at day 28, compared with the control-group at day 28 (Fig. 2).

### Postoperative complications and macroscopic findings

No anastomotic leakage occurred. Five piglets developed an incisional hernia, and seven piglets minor incisional infection. Both complications were equally distributed between the two groups and caused no intervention. All piglets were thriving clinically as assessed by the Piglet Grimace Scale [20]. Visual assessment during the autopsies revealed dilated reversed segment in 7 of 11 piglets in the intervention group, but the degree or extent was not measured. In several piglets, the dilation reached beyond the proximal anastomosis. No dilation of intestine was observed in the control group.

## Discussion

This study showed, that reversal of a 25 cm long segment of the distal small intestine in our SBS model with excision of 70% of the distal small intestine had a negative effect on weight gain compared to control at a 28 days follow-up. Our results are in concordance to some studies [21–25] and different from others [26].

In a similar short bowel model with excision of 60% of the distal small intestine length and reversal of 20 cm of the distal small intestine, the weight gain in the group with a reversed segment was 2.31 kg at one month follow-up compared to 2.03 kg in the control group. The difference was not significant [21]. In another model, small intestine from 150 cm distal to the ligament of Treitz and 150 cm proximal from the ileocecal valve was excised. Two weeks later a Bianchi procedure was performed in one-third of the animals, reversal of the distal 10 cm of jejunum in another one-third while the rest served as a control group. A similar weight gain of approximately 20 kg was found in the three groups after a total of 8 weeks, compared to 40 kg in shamed operated with no intestinal resection. Malnutrition measured by fecal fat loss and serum albumin occurred in all three intervention groups [22]. In the SBS model by Digalakis et al. [23] performed in piglets 12–14 weeks of age, the small intestine was excised from 100 cm anally from the ligament of Treitz to 100 cm orally from the ileocecal valve, equalling 80%. In the intervention group a 28–30 cm segment of the distal jejunum was reversed, and at postoperative day 60, no significant differences in weight gain were found between the two groups.

It is of great importance to identify the necessary extent and location of small-bowel excision to induce short bowel syndrome in an experimental animal model. It may depend on the type of animals used, whether they are full-grown or not, and on the anatomic location of the remaining small intestine.

A universal definition of SBS in animal models is lacking. In a study by Frongia et al. [27] three groups of porcines (n = 5 in each group) 75, 90 and 100% of small bowel was resected and in all groups a weight loss was observed at postoperative day 14. Not surprisingly the weight loss was highest in the group with 100% resection, but the differences were not statistically significant. The authors suggested that a 100% small bowel excision should be applied for acute short term experiments, and a 75 or 90% excision for studies with longer observation periods. A systematic review by Weih et al. [28] showed that the amount of resected small bowel varied from 75 to 100% to induce SBS, but they also pointed out that that it is fundamental to tailor the SBS-model according to the aim of the study.

For the approval from The Animal Experiments Inspectorate a maximum of the resected small intestine was set to 70%. Our project was considered a long-term experiment. Whether a 5% greater resection would have changed the results are unknown. Furthermore, there is an ethical limit for the percentage of bowel that can be excised. Thus, most studies regarding segmental reversal report removal of 60–80% of the small intestine [21–24, 26], which was enough to induce SBS in some studies, but insufficient in others. In our study, the removal of 70% of the small intestine and reversal of a 25 cm distal jejunal segment showed a significant difference in weight. When compared to an expected average weight gain of approximately 13 kg/month in healthy animals, this raises the question whether an SBS model has been established [29].

This length of the reversed segment was chosen from the available literature [21–23]. In the study by Grave et al. [21] 20 cm of distal small intestine was reversed without any significant differences in weight gain. In the study by Digalakis et al. [23] a 80% mid intestinal resection was performed leaving 100 cm of proximal jejunum and distal ileum, respectively. A 28–30 cm long intestinal reversal was performed on the proximal jejunal remnant. This is very uncommon in relation to the clinical situation, where a reversal of the distal small intestine is preferred and most relevant because most of the reversals performed in humans are in relation to stoma reversal with an intestino-colonic anastomosis. This will leave the patient with two anastomoses only as opposed to three anastomoses by choosing a more proximal reversal. Finally, the absorbable capacity of the small intestine increases distally. The study by Digalakis et al. [23] showed histopathological changes suggestive for an enhanced intestinal adaptation without unfavorable influence on the intestinal transit time. This was the reason why we choose the reversal of a 25 cm long segment of the distal jejunum fully aware that the length of the reversed segment is a balance between maximal intestinal absorption capacity and functional obstruction.

One limitation of our study is the short four weeks follow-up. Even though the follow-up period is short, the difference in weight gain was statistically significant. The lacking measurement of individual food-intake and faecal output may be another limitation.

Some SBS models uses a fixed length of the remnant small intestinal segment [22, 23, 27]. This could be a confounder as the length of the small intestine is dependent upon body weight [30].

The estimation or calculation of the fractional length of the resected small intestine is a problem. In our study, we measured the total length of the small intestine in each piglet prior to resection, which is difficult and may be subjected to sources of errors as demonstrated by a wide range of measurements between animals (550–1185 cm). Some of the explanation might be differences in the weight of the animals. Resection of different parts of the small intestine may be of importance for SBS models. Excision of the ileocecal valve results in a more rapid gastrointestinal transit time, and the segmental reversal procedure could theoretically be more beneficial.

In this study the induction of SBS and the surgical intervention was performed simultaneously, which leaves no time for intestinal adaptation to the SBS. This is contrary to the human studies, where the reversal procedure was performed months to years after development of SBS [7].

At autopsy, we found that several piglets in the intervention group had dilated small intestine indicating the presence of a chronic subileus condition, although no further data of the extent of dilation is available. The subileus may explain the lower weight gain in the intervention group and also to some extent the lesser crypt depth and villus height in the intestine, proximal to the reversed segment found at the histological examination. Findings in other studies are contradictory [21, 23]. With the difference in villus height and crypt depth one would have expected a similar difference in the ki-67 expression. However, no significant differences were found, similar to the results found by Grave & Thomsen et al. [21].

GLP-2 and GIP was measured as a marker of the secreting capacity of respectively colon and proximal intestine. In the intervention group, there was a tendency to decreased plasma concentration of both GLP-2 and GIP from day 0 to day 28, whereas a significant increase was found in the control group, which might reflect the intestinal hypertrophy with a greater villus height in the control group. The findings with lower villus height, lesser crypt depth and lower GIP-levels in the intervention group might indicate, that the reversed segment was too extensive.

As opposed to the findings in the study by Digalakis et al. [23] we found a lower villus height and crypt depth in the intervention group which may explain the smaller weight gain in this group. The absent increase in GLP-2 in the intervention group might indicate an impaired intestinal adaptation [31] and the absent increase in GIP as a result of chronic obstruction in the revised segment [32].

Other accepted surgical approaches to autologous intestinal reconstruction has been developed with intestinal lengthening a.m. Bianchi or STEP procedures [33]. A number of criteria such as minimal remnant intestine length, presence of intestinal dilation and preserved ileocecal valve is of importance before considering surgery [33]. SILT (Spiral intestinal lengthening and tailoring) may be a promising alternative but needs further comparative studies before implementation [34, 35]. If autologous intestinal reconstruction is not possible or unsuccessful, or the patient develops serious complications to PN, intestinal transplantation remains a possibility [36].

The most extensive study that has been published regarding the clinical effect of the segmental reversal procedure in SBS is from Layec et al. [37]. Seventeen patients who had a reversal of distal small intestine were matched to 17 control patients. Intestinal absorption was significantly higher for a number of essential nutrients in the reversal group. During an observation period of median 16 (6–132) months, 67% could be weaned of home parenteral nutrition compared to 52% in the control group, after a median observation period of 47 (6–227) months. Several case reports (follow-up 1–60 months) regarding the segmental reversal procedure have been published [38–40], and the majority reported beneficial results regarding intestinal absorption and weaning of HPN. The positive results in human studies are difficult to compare to an animal model. Most studies are performed on healthy animals, with short observation periods, where intestinal adaptation has not reached its maximum. Our study showed that a too long segment for reversal might have a detrimental effect.

## Conclusions

Treatment of short bowel syndrome with a reversed jejunal segment of 25 cm in our pig model had a detrimental effect on the weight gain.

## Data Availability

The datasets during and/or analyzed during the current study available from the corresponding author on reasonable request.

## Abbreviations

SBS: Short bowel syndrome
GLP-2: Glucagon-like peptide-2
GIP: Glucose-dependent insulinotropic peptide
SD: Standard deviation
PN: Parenteral nutrition
HPN: Home parenteral nutrition
STEP: Serial transverse enteroplasty
SILT: Spiral intestinal lengthening and tailoring1
